# Comparing the risk of low-back injury using model-based optimization: Improved technique versus exoskeleton assistance

**DOI:** 10.1017/wtc.2021.12

**Published:** 2021-10-01

**Authors:** Giorgos Marinou, Matthew Millard, Nejc Šarabon, Katja Mombaur

**Affiliations:** 1 Optimization, Robotics and Biomechanics (ORB), Institute of Computer Engineering (ZITI), Heidelberg University, Heidelberg, Germany; 2 Faculty of Health Sciences, University of Primorska, Izola, Slovenia; 3 Canada Excellence Research Chair in Human-Centred Robotics and Machine Intelligence, Systems Design Engineering & Mechanical and Mechatronics Engineering, University of Waterloo, Waterloo, Ontario, Canada

**Keywords:** back-support exoskeletons, low-back pain, model-based optimization, optimal control, wearable robotics

## Abstract

Although wearable robotic systems are designed to reduce the risk of low-back injury, it is unclear how effective assistance is, compared to improvements in lifting technique. We use a two-factor block study design to simulate how effective exoskeleton assistance and technical improvements are at reducing the risk of low-back injury when compared to a typical adult lifting a box. The effects of assistance are examined by simulating two different models: a model of just the human participant, and a model of the human participant wearing the SPEXOR exoskeleton. The effects of lifting technique are investigated by formulating two different types of optimal control problems: a least-squares problem which tracks the human participant’s lifting technique, and a minimization problem where the model is free to use a different movement. Different lifting techniques are considered using three different cost functions related to risk factors for low-back injury: cumulative low-back load (CLBL), peak low-back load (PLBL), and a combination of both CLBL and PLBL (HYB). The results of our simulations indicate that an exoskeleton alone can make modest reductions in both CLBL and PLBL. In contrast, technical improvements alone are effective at reducing CLBL, but not PLBL. The largest reductions in both CLBL and PLBL occur when both an exoskeleton and technical improvements are used. While all three of the lifting technique cost functions reduce both CLBL and PLBL, the HYB cost function offers the most balanced reduction in both CLBL and PLBL.

## Impact Statement

Injury to the low back is common among workers, painful to individuals, and costly to society as a whole. Two risk factors associated with low-back injury are the cumulative low-back load (CLBL), and the peak low-back load (PLBL). In this work, we use simulation and a two-factor block study design to examine how much the risk of low-back injury is affected by an exoskeleton and improvements to lifting technique. Our simulations indicate that training alone can substantially reduce CLBL, but only modestly reduce PLBL. When both training and an assistive exoskeleton are used, our simulations indicate that both risk factors can be substantially reduced.

## Introduction

Low-back pain (LBP) accounts for approximately 15.5% of worker absenteeism in industry across Europe and North America (Wynne-Jones et al., 2014), while being globally the first ranked disorder for years lived with disability (Buchbinder et al., 2013). Assistive exoskeletons, and more specifically, back-support exoskeletons, are being developed for the prevention of LBP by alleviating low-back loads. There exists a variety of passive exoskeletons such as Laevo (Laevo, 2018; Hensel and Keil, 2019), Personal Lift Assist Device (PLAD) (Abdoli-E et al., 2006), SuitX (Kazerooni et al., 2019), Moment Restoring Device (Wehner et al., 2010), and Bending Non-Demand Return (BNDR) (Ulrey and Fathallah, 2013). There are a similar number of active exoskeletons such as the Robomate (Huysamen et al., 2018; Toxiri et al., 2018), Wearable Stop-Assist Device (WSAD) (Luo and Yu, 2013), and the Muscle Suit (Muramatsu and Kobayashi, 2014). In many cases, these devices manage to lower joint torques and muscle activity (de Looze et al., 2016). Although many exoskeletons are focused at reducing the risk of low-back injury, it is not clear how effective these devices are in comparison to simply improving lifting technique.

Repetitive lifting and bending tasks contribute most to LBP (Coenen et al., 2013). Repetitive lifting causes the accumulation of microdamage to the tissue through cumulative low-back loads (CLBLs; Brereton and McGill, 1999). Apart from CLBL, instantaneous damage can be caused to the lower back by peak low-back loads (PLBLs). Both of these quantities are typically highest at the L5/S1 lumbosacral joint (Coenen et al., 2014). Risk factors based on the L5/S1 extension moment (such as CLBL and PLBL) are both easy to calculate and capture the risk associated with many different specific injuries, since the loads applied to the ligaments, disks, vertebrae, and muscles of the back, scale with the L5/S1 extension moment (van Dieën, 2005).

Even though there has been a lot of modeling and simulation work done to learn more about back injury (McGill and Norman, 1987; de Zee et al., 2007; Christophy et al., 2012), much of this work is based on inverse-dynamics data taken from real people lifting, and therefore cannot predict how someone might use a novel exoskeleton. The few optimal control studies that have been done to predict new lifting motions (Xiang and Arefeen, 2020) do not combine human and exoskeleton models. The limited amount of work that includes an exoskeleton (Millard et al., 2017; Harant et al., 2019), however, does not consider the effects of training, nor risk factors related to low-back injury. In addition, no simulation work has been found that examines the effect of lifting technique on the risk of low-back injury.

In this work, we employ optimal control and multibody dynamics to model, simulate, and predict the movements and forces needed for a person to lift a 10-kg box from the floor. Using motion capture data of an experimental participant lifting a 10-kg box from the floor, we recreate the motion using a least-squares (LSQ) fitting approach. We then add the exoskeleton model and re-evaluate the LSQ problem in order to quantify the effect of adding an exoskeleton without changing the original motion. Next, we examine how much CLBL and PLBL can be reduced through improved lifting technique, with the use of an exoskeleton, and lastly with the use of an exoskeleton plus improved lifting technique. In this study, we use a movement between a stoop and a squat (stoop–squat), since this technique is most often used when picking up objects from the ground (Burgess-Limerick et al., 1995). We extend our previous work (Marinou and Mombaur, 2020) by simulating the effects of two different conditions in our simulations: lifting using experimentally measured technique versus lifting with optimal lifting technique; and lifting without any aids versus lifting with the assistance of an exoskeleton. We evaluate three different optimal lifting techniques that minimize three different risk factors for lifting: (a) CLBL, (b) PLBL, and (c) a weighted sum of both CLBL and PLBL (HYB). We evaluate the effects of assistance by simulating the lift using two different models: a model of our human participant, and a model of our human participant wearing the SPEXOR exoskeleton. We hypothesize that both the lifting technique alone and with the exoskeleton will reduce CLBL and PLBL, but that the exoskeleton will provide the greatest reduction in injury risk.

## Methods

**Figure 1.**
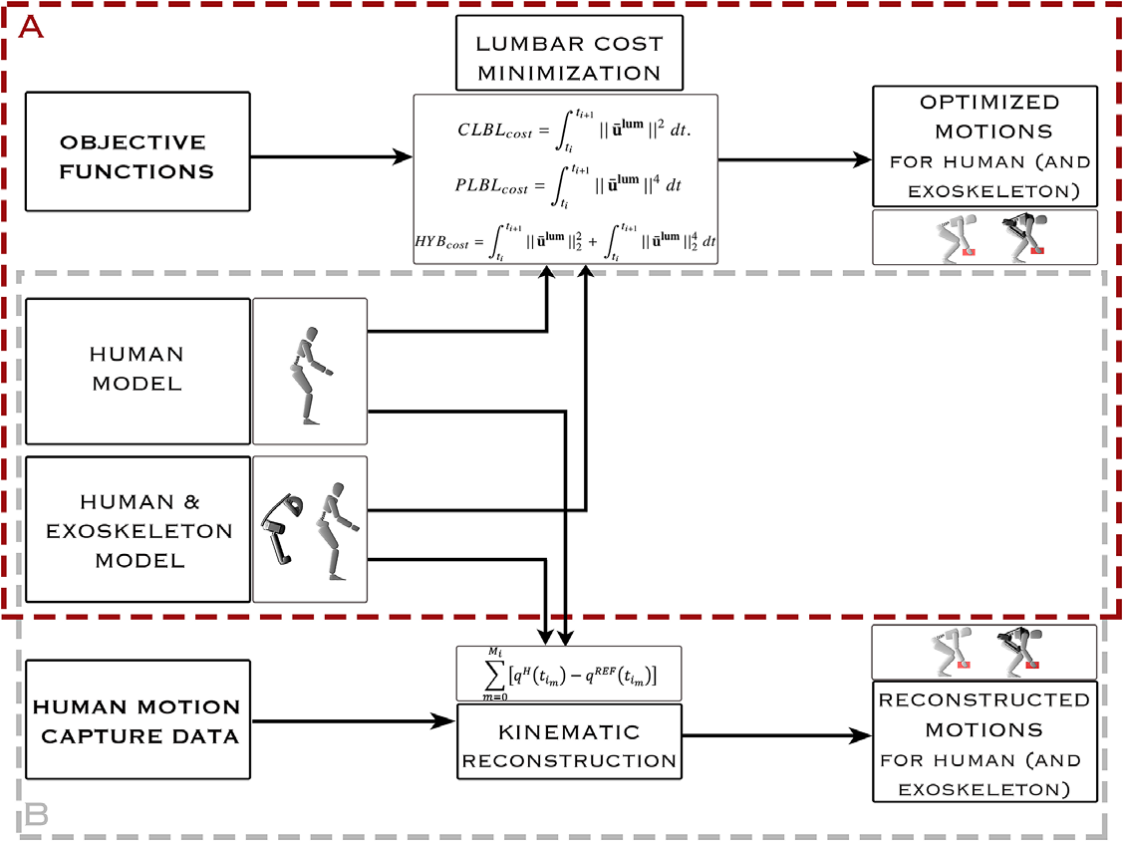
Two optimization methods: (a) prediction through optimization of human-only (HO) and human-with-exoskeleton (HwE) stoop–squat lifts and (b) dynamic reconstruction of human-recorded stoop–squat lifts for both HO and HwE optimal control problems based only on human capture data.

We simulate eight different stoop–squat lifts of a 10-kg box in the sagittal plane using a planar multibody model. We create a reference solution using an LSQ fitting problem (Figure 1b), where we track the motion of one participant from the recorded motion capture data. To examine how much an exoskeleton reduces the risk of injury using the same lifting technique, we solve the same LSQ fitting problem but using a model that includes the exoskeleton. The remaining six of the lifts are completely synthesized and rely on no experimental data (Figure 1a). To examine the effect of lifting technique, we create three different cost functions: one for minimizing CLBL, one for minimizing PLBL, and lastly a hybrid function minimizing both CLBL and PLBL. The following sections describe our modeling procedure, the optimal control problem (OCP) formulation, and the evaluation of the cost functions employed.

### Experimental Data

A male participant of 76.5-kg mass and height of 1.69 m performed a stoop–squat lift of a 10-kg box. Kinematics of the body segments and the box were recorded by recording the position of markers on the participant and the box, using an OptoTrack system (Northern Digital Inc., Waterloo, Ontario, Canada). Ground reaction forces were collected using Kistler force plates (model 9260AA6 from Kistler, Winterthur, Switzerland). The experiment was conducted at the University of Primorska in Slovenia and approved by the national medical ethics committee of the Republic of Slovenia (0120-199/2016-2, KME 93/04/16) with written and informed consent from the participant. The data were collected for the human alone, without the aid of the exoskeleton.

### Dynamics

We have modeled the human body in the sagittal plane using an 11-segment model with 13 degrees-of-freedom (DOFs), the exoskeleton with 6 segments and 8 DOFs, and the box with 1 segment and 3 DOFs. Accordingly, the generalized position vector contains 3 entries for the box, and 13 entries for the human model. When the exoskeleton is included, eight additional entries are added for the exoskeleton model. The geometry, masses, and inertias of the segments in the human model have been scaled using de Leva’s (1996) anthropomorphic tables and the participant’s height and mass. The mass and geometry properties of the box and the exoskeleton have been set to match the physical box used in the experiment and the SPEXOR exoskeleton (Näf et al., 2018).

**Figure 2.**
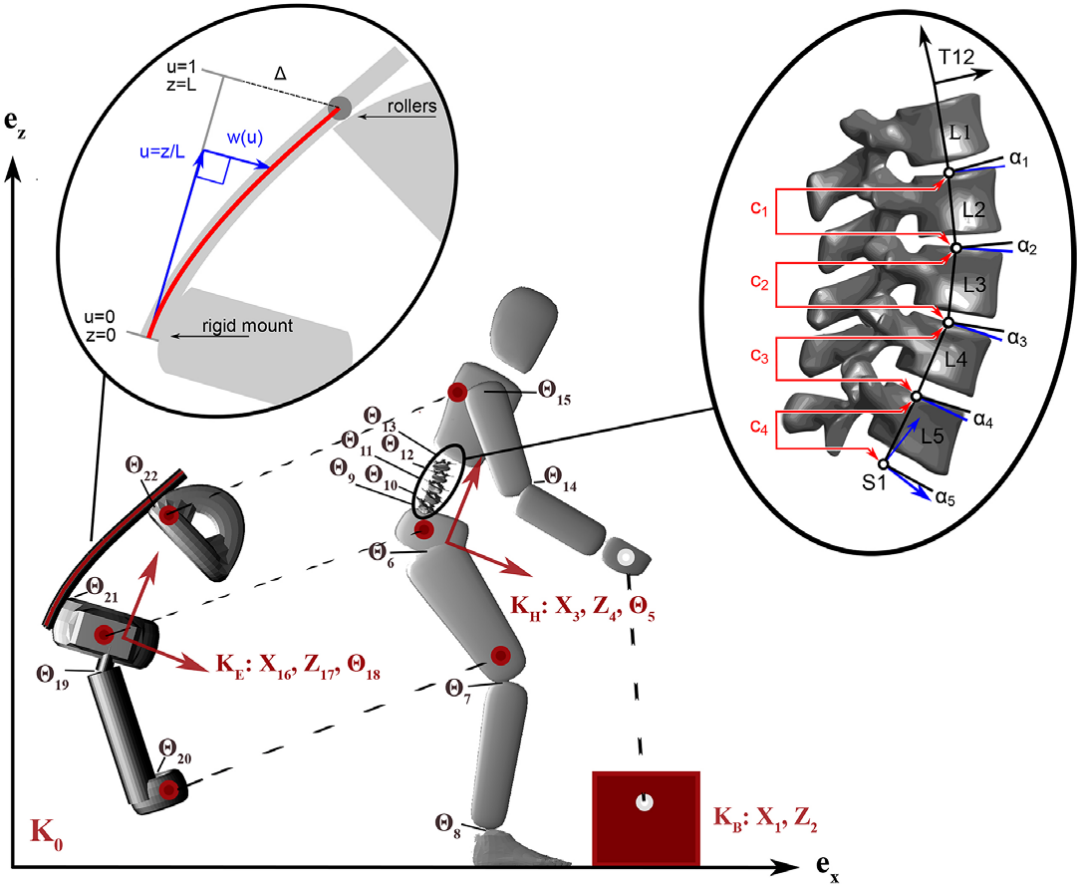
The human as an 11-segment, 13-DOF model and the attachment points to the 6-segment, 8-DOF exoskeleton. Dashed lines indicate the kinematic constraints between the exoskeleton and the human, as well as the human and the box. The feet are constrained to the ground throughout the motion, whereas the box is constrained to the ground until lifted by the human. The letter 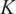 denotes a coordinate system where the subscripts 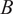 , 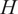 , 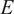 , and 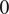 correspond to the coordinate systems of the box, human, exoskeleton, and global reference frames, respectively. The planar positions are indicated with 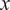 and 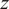 and angles by 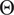 . A close-up of the lumbar spine model depicts the L1–L5 lumbar vertebrae and the four constraint equations that couple the movements of the joints. Each disk is approximated as a spherical joint located at the center of rotation identified by Pearcy and Bogduk (1988) from radiographic data. We have scaled the center of rotation of each vertebrae to fit the high-resolution meshes of the lumbar vertebrae of Mitsuhashi et al. (2009).

The system is modeled as a constrained multibody system,
(1)

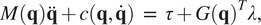

where 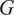 is the Jacobian of the kinematic constraint equations
(2)

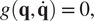

and 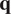 , 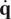 , and 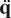 are the generalized vectors for position, velocity, and acceleration of the model’s segments. 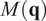 is the mass matrix of the system, and 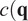 , 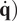 is the vector of Coriolis and centripetal forces. Equation (2) describes the kinematic constraints, which consists of the coupling equations for the lumbar spine (Christophy et al., 2012), the contact constraints between the foot and the ground, the contact constraints between the hands and the box, and the constraints that attach the exoskeleton to the human body model. The entries contained in 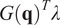 are the generalized forces imposed by these constraints, where 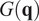 is the Jacobian of the constraint equations 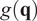 with respect to 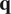 , and 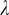 is a vector of Lagrange multipliers. Finally, 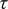 is the vector of generalized force applied to the system, which consists of the joint torques developed at the internal joints of the human model and the hip actuator of the exoskeleton (Figure 2).

In describing the interaction between the human and the exoskeleton, we simulate the exoskeleton as an external rigid body which is attached to the human through eight kinematic constraints defined at three attachment points: the thigh, pelvis, and upper trunk. The thigh and upper trunk modules of the exoskeleton are attached to the human model using weld constraints. A point constraint is used to attach the pelvis module of the exoskeleton to the pelvis, which permits rotation between these two bodies. Weld constraints are applied between the human feet and the ground, as well as the hands and the box during the contact and lifting phases. To simulate this constrained system forward in time, we use the Rigid Body Dynamics Library of Felis (2017).

### Lumbar Spine Model

We have included an articulated and coupled model of the lumbar spine similar to Christophy et al. (2012) to ensure that the bending movements of the model are as accurate as possible. In this model, the lumbar spine is described as five vertebrae attached serially using revolute joints. The revolute joints of the lumbar back are located at the average center of rotation of each vertebra as reported by Pearcy and Bogduk (1988). We have fit the data of Pearcy and Bogduk (1988) to the high-resolution vertebral meshes of Mitsuhashi et al. (2009), and scaled the model to fit our participant. All internal joints of the human model are torque-driven.

Although the lumbar spine has five joints, we have coupled those joints with four constraint equations, so that the entire lumbar spine has only one DOF in the sagittal plane. The constraint equations have been formulated, so that the resulting coordinated motion is consistent with the coordinated bending of the lower back as measured by Wong et al. (2006). Wong et al. (2006) observed that the flexion of each joint, 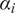 , scales linearly with the total lumbar flexion angle, 
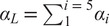
 , such that 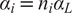 , where 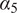 corresponds to the angle from S1 to L5, and 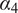 corresponds to the angle from L5 to L4, and so forth. The coefficients 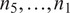 that best fit the 30 participants in Wong et al.’s (2006) study are 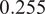 , 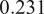 , 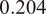 , 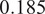 , and 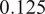 . Similar to Christophy et al. (2012), we use the linear relationship between 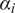 and 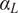 to form the velocity-level constraint
(3)

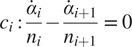

between neighboring pairs of joints (Figure 2). Prior to simulation, each lumbar joint angle is biased, so that a lumbar flexion angle of zero, 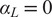 , poses the lumbar spine to match the resting position (as shown in Figure 2) of the participant from Mitsuhashi et al. (2009). In this case, the bias angles are 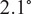 , 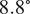 , 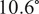 , 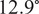 , and 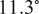 of extension for the joints from the L5/S1 joint to the L1/L2 joint, respectively. As with other kinematic constraints in this model, index reduction is used to transform the original set of differential algebraic equations of Index 3 to a system of differential algebraic equations of Index 1. During the simulation, the constraint error is reduced using the stabilization of Baumgarte (1972).

### Exoskeleton Model

We model the SPEXOR exoskeleton using an eight-DOF mechanism composed of six segments and a total mass of 9.12 kg. Unique to this exoskeleton are three carbon fiber rods (4.7 mm in diameter, with a Young’s modulus of 166 GPa) producing counter torques about the lumbar in order to support lifting motions. In addition, a hydraulic actuator (with a maximum output torque of 25 Nm) is placed at the exoskeleton hip joints, which feature a misalignment compensation mechanism (Figure 2). The exoskeleton further includes a trunk and pelvis module that are connected by carbon fiber beams, and two thigh modules that are connected to the pelvis interface by a rigid metal rod on each side. The beams are rigidly fixed to the pelvis module and pass through the torso module via a series of rollers.

We model the path traced by the slender beams using a cubic spline. As has been shown by Holladay (1957), a cubic spline traces a path that minimizes total curvature, which is the same path traced by a slender elastic beam. At every instant in time, a cubic spline 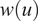 is fitted to match the end conditions imposed by the rigid pelvis mount and the rollers (Figure 2). We describe the spline in normalized coordinates
(4)

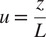

along the undeformed path of the beam, and by deflections
(5)

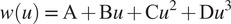

perpendicular to 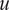 . Here, 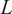 is the distance between the pelvis mount and the rollers projected onto the beam’s axis that is fixed to the pelvis module. The coefficients in Equation (5) are evaluated using the boundary conditions imposed by the rigid pelvis mount
(6)

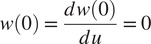

and the boundary conditions imposed by the rollers on the torso module where the beam must deflect by 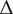



(7)

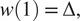

and
(8)

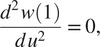

since the rollers cannot apply a reaction moment. The moments and shear forces the slender bent beam applies to the pelvis ( 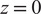 ) and torso ( 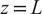 ) modules are evaluated using the spline 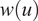 and an Euler–Bernoulli beam model where
(9)

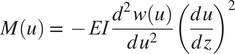

and
(10)

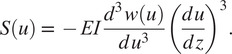




### OCP Formulation

**Figure 3.**
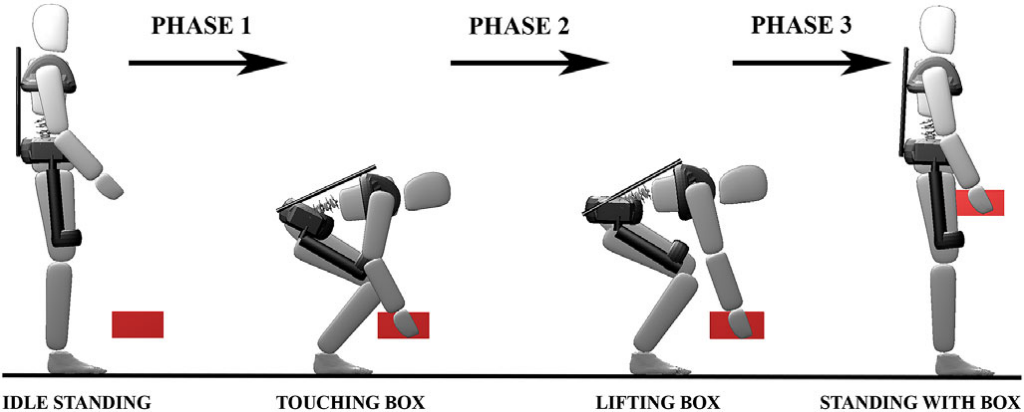
We formulate lifting as a three-phase problem: standing to touching the box, touching the box to lifting the box, and finally lifting the box to standing back up again with the box. Image sequence taken from the LSQ HwE optimal control problem.

We divide the task of lifting a box from the floor into three phases for both human-only (HO) and human-with-exoskeleton (HwE) OCPs (Figure 3): first, the model moved from a standing phase to touching the box; second, the model applies force to the box until the full weight of the box is supported; finally, the model lifts the box and stands back up again. We use a series of constraints to ensure that contact forces are physically realistic and the beginning and end poses are comparable. The forces in the global 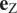 direction are constrained to be positive, while friction forces must be within the friction cone. Similarly, tangential forces between the hands and the box are also constrained to be within the friction cone, as would be case if the lift is being performed with an open grip. Finally, we constrain the model to begin and end the lift at rest and in the same starting and ending pose as the human participant.

For the simulations that include an exoskeleton, we have additional constraints to limit the interaction forces between the human and the exoskeleton. We solve both problems using a direct multiple-shooting algorithm implemented in MUSCOD-II (Bock and Plitt, 1985; Leineweber et al., 2003).

In this work, we formulate a multiphase OCP that minimizes the Lagrange term
(11)

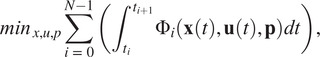

which includes the control vector 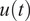 which is composed of the joint torques of the human model and the actuator control signal of the exoskeleton, and the state variables vector 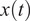 which contains the positions and velocities of the multibody system segments. The vector 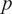 stands for physical parameters, such as exoskeleton, and 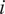 iterates through the multiple phases of the problem through time *t* from 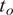 to 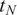 . The dynamics of the systems (Equations (1) and (2)) take the form of ordinary differential equations
(12)

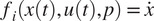

that are limited by equality and inequality constraints
(13)

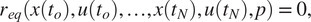



(14)

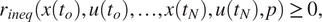

at specific time points throughout the separate phases. The time vector is broken up into *N* consecutive time intervals
(15)

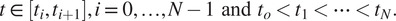




#### *LSQ quadratic* *fitting*

To find solutions that mimic the human participant’s lifting technique, we solve an LSQ problem. For both HO and HwE formulations, the LSQ problem has two terms: a tracking term
(16)

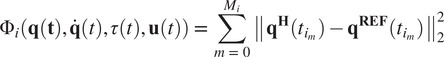

and a term
(17)

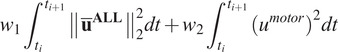

to ensure that the exoskeleton is used during the HwE simulations. We include a small regularization on the exoskeleton’s motor torque, so that the exoskeleton is not unnecessarily used. 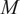 denotes the number of shooting nodes for the given phase 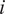 , and 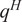 and 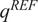 are the computed and tracked positional coordinates, respectively. The vector 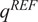 is a function of time that comes from using inverse kinematics to pose the model, so that its virtual markers minimize the squared distance to the recorded positions of the markers on the participant. The vector 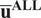 is the normalized human joint torques vector, and 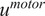 is the control signal of the motor which is used as a regularization term. 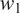 and 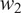 have the values of 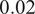 and 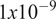 , respectively.

#### Synthesizing lifting techniques with a lower risk of injury

We synthesize three lifting motions that minimize the risk factors associated with low-back injury: CLBL, PLBL, and a weighted sum of both CLBL and PLBL. When analyzing experimental data, the CLBL is calculated as
(18)

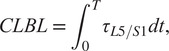

as described by Coenen et al. (2013). Although Equation (18) is perfectly suited for analysis, it is ill-suited as a cost function for an OCP, because it is signed: it is possible for the model to produce a flexion torque and thus reducing the CLBL after lifting. Instead, we integrate the normalized torque of the lower back squared
(19)

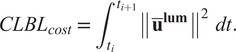




Squaring the lumbar torques has been suggested by Coenen et al. (2012) to put a bit more emphasis of the CLBL result on higher loads. We have also normalized these values according to the maximum torque output of these joints ( 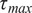 ), so that their value remains between 0 and 1, to avoid numerical scaling problems that occur with either very big or very small numbers. Since the motion of the five vertebrae are coupled by four constraint equations, it is possible to drive the lumbar spine using only a subset of the joints, but this is physiologically unrealistic. By summing across the moments developed by each joint, we ensure that the final load distribution does not favor one vertebra at the expense of the other joints.

When analyzing experimental data, the PLBL is evaluated as
(20)

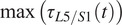

over a time duration of interest (Coenen et al., 2012). Both Coenen et al. and Jäger et al. (2013) have suggested instead to evaluate PLBL risk by integrating the L5/S1 moment over time but raised to a higher power to further penalize peak values. To find motions that reduce PLBL risk, we minimize the sum of normalized lumbar torques
(21)

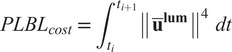

raised to the power of 4.

Finally, we consider a third cost function which is simply the sum of these two cost functions
(22)

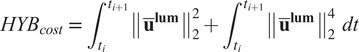

in hopes of finding a motion that is able to reduce both risk factors simultaneously. In all cost functions, we include a small regularization term that includes all joint torques
(23)

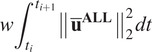

 to ensure that the result is a minima (Nagarajan and Kolter, 2017). The weight factor 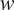 takes the value of 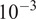 .

### Evaluation

We use the following procedure to evaluate our results:

- We compare the peak lumbar load and maximum lumbar flexion angles of our participant to the values reported in the literature (Kingma et al., 2004) where 10 participants performed a stoop–squat lift with a 10.5-kg box.
- We report CLBL and PLBL which we have calculated according to Coenen et al. (2013), for each of the prediction simulations and dynamic reconstruction problems.
- We report the results for the four study blocks: HO with tracking (LSQ); HO with improved lifting technique; HwE with tracking; and HwE with improved lifting technique.

## Results

**Figure 4.**
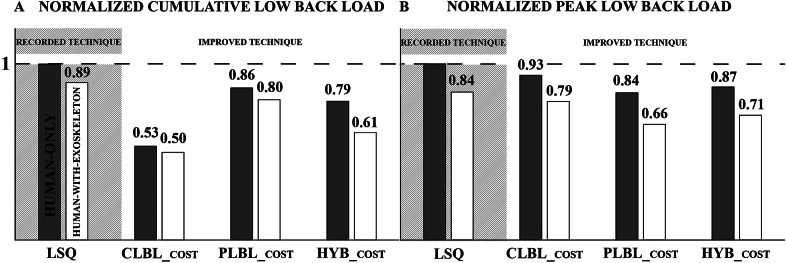
Bar plot representing normalized lumbar torques about the L5/S1 joint from simulations of (a) cumulative low-back loads and (b) peak low-back loads. Shaded bars refer to HO and white bars to human-with-exoskeleton optimal control problems (OCPs). The torques are normalized according to the L5/S1 joint torque from the result of the HO tracking (LSQ) OCP. Numbers on top of bars indicate the values of the normalized torque, relative to the HO LSQ of value 1.

**Figure 5.**
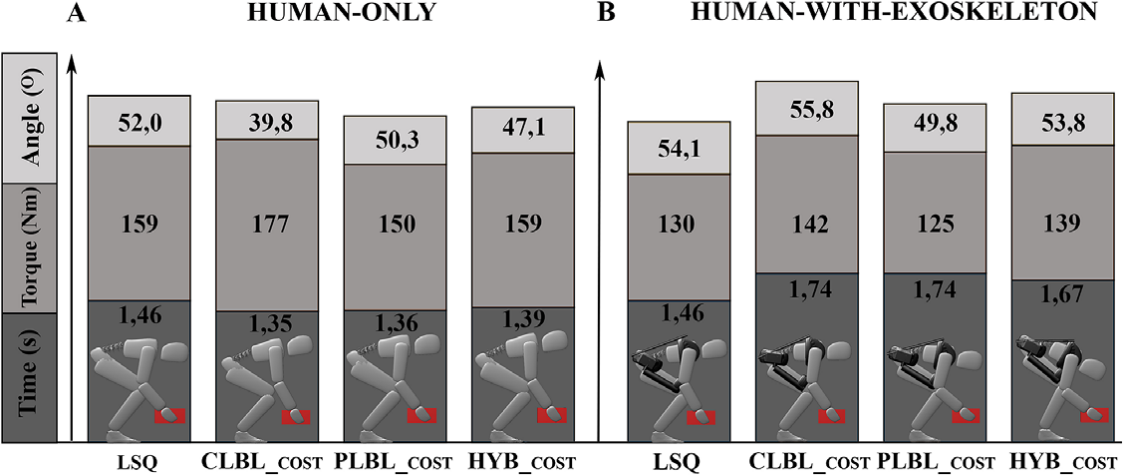
Models of (a) human only and (b) human with exoskeleton at the moment of lifting the box for all objective functions. The shaded regions in the background resemble a stacked bar plot, indicating the time (seconds) of box liftoff with the respective peak torques (Newton meters) and lumbar flexion angles (degrees) achieved at the point of lifting the box, for every model separately, with all the values indicated in the respective boxes.

**Figure 6.**
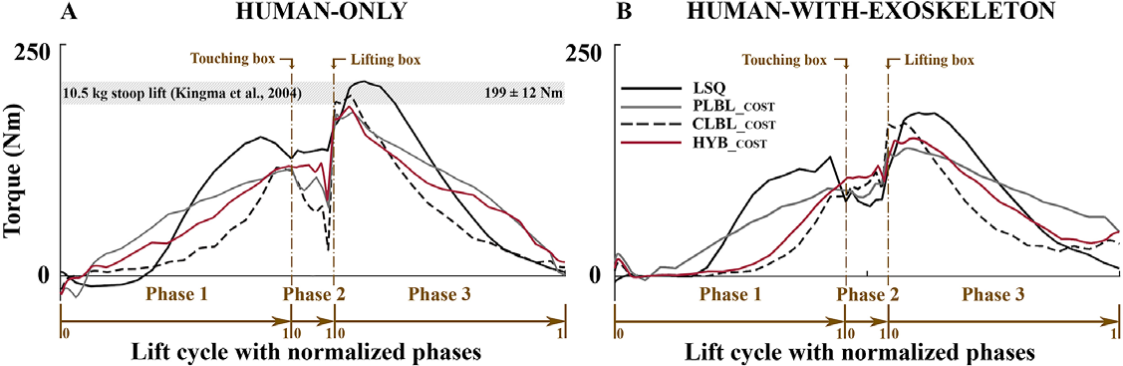
L5/S1 torques for (a) human only (HO) and (b) human with exoskeleton as calculated for the biomechanic metrics of cumulative low-back load and peak low-back load. The shaded region in the HO plot reports the values measured in literature (Kingma et al., 2004) for net lumbar torque. The phases of the minimization problems were scaled according to the experimental phases for easier graphical comparison.

Simulated improvements to lifting technique were able to reduce the CLBL and PLBL risk factors for the unassisted lifts (Table 1 and Figures 4, 5a, and 6a). The experimental participant performed a stoop–squat lift with a peak torque of 210 Nm and a cumulative torque of 243 Nms (Table 1 and Figure 5) according to the dynamic reconstruction, close to the peak torque range reported by Kingma et al. (2004) of 199 ± 12 Nm. These simulation results indicate that training can produce modest reductions in PLBL, and larger reductions in CLBL (Table 1 and the gray bars in Figure 4).

**Table 1.** Cumulative low-back load (CLBL) and peak low-back load (PLBL) torques for both human-only and human-with-exoskeleton simulations for the four different optimal control problems (OCPs). The least-squares (LSQ) entry is the reference used for comparison purposes, by tracking the motions of the experimental participant

OCP	CLBL (Nms)		PLBL (Nm)	
	Human only	Human with exoskeleton	Human only	Human with exoskeleton
LSQ	243	217	210	177
*CLBL_cost_*	130	121	195	165
*PLBL_cost_*	210	193	176	138
*HYB_cost_*	190	148	182	148

*Note.* CLBL and PLBL have different units, as CLBL is the torque as a function of time, whereas PLBL is the torque at a specific point in time. All values rounded to three significant figures.

Larger reductions in both CLBL and PLBL can be observed in the HwE OCPs. While it was not possible to reduce the PLBL in the HO simulation by more than 16.2%, the exoskeleton was able to achieve a 34.3% reduction (Table 1 and the white bars in Figure 4) when looking at the *PLBL_cost_* metric. In addition, the exoskeleton was able to reduce further both CLBL and PLBL in all cases when compared with the HO simulations (Figure 6). While the overall trend in torques for the various solutions appears to be similar in both unaided and aided motions, Phase 2 does not follow this pattern. The drop in torque during the HO simulations at the end of Phase 2 (Figure 6) happens as a result of a rapid counter movement: just prior to lifting the box, the model relaxes and drops the hips. During the exoskeleton assisted lifts, the model does not exploit this technique presumably, because the exoskeleton’s support makes this counter movement less effective.

**Figure 7.**
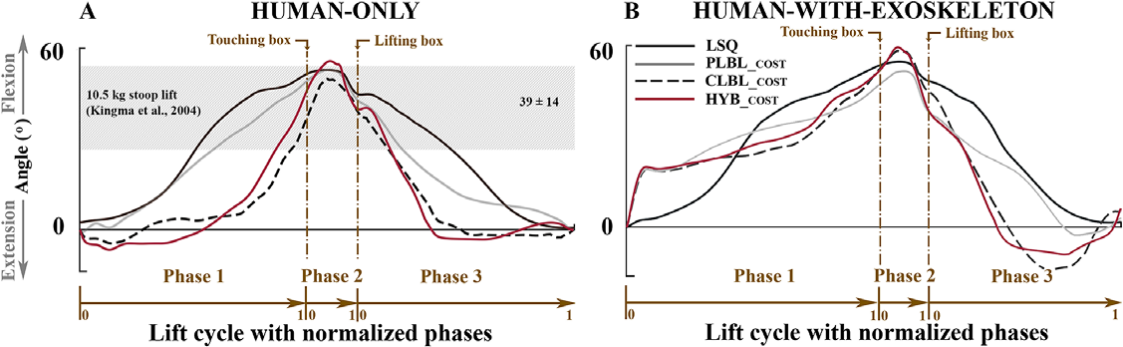
Lumbar flexion angles for (a) human only (HO) and (b) human with exoskeleton as resulted from the tracked and optimized stoop–squat motion. The shaded region in the HO plot reports the values measured in literature (Kingma et al., 2004) for net flexion angle. The phases of the minimization problems were scaled according to the experimental phases for easier graphical comparison.

**Figure B1.**
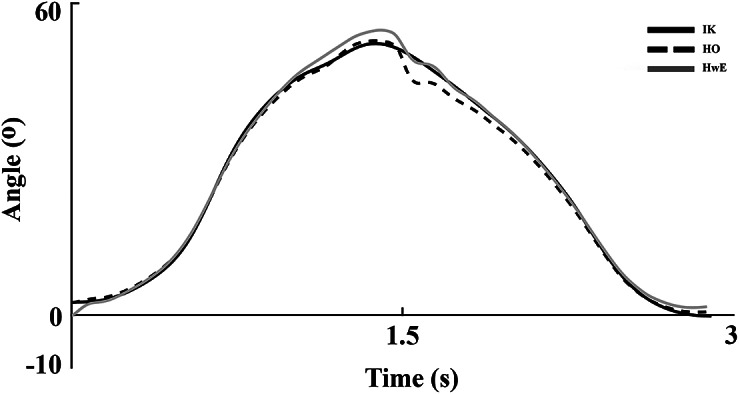
Lumbar flexion angles for the inverse-kinematics (IK) solution obtained from motion capture versus human-only (HO) least-squares optimal control problem (OCP) and human-with-exoskeleton (HwE) solutions from our OCPs. This serves as a validation of our dynamic reconstruction procedure, so as to show the close correlation between our solution and the IK data from the motion capture.

The lumbar flexion angle closely tracked the participant data in the LSQ solution (Figure B1 in Appendix B) and was substantially reduced when the model was free to move. The dominant factor for the peak lumbar flexion angle appears to be the cost function, as there is little difference between the lumbar flexion angles of the HO and HwE lifts. Curiously, all the minimization cost functions for the HO condition result in a lower lumbar flexion angle in Phases 1 and 2 (Figure 7), whereas for the same phases in the HwE condition, the exoskeleton seems to be increasing the lumbar flexion angle of the human. In addition, we can see that the *CLBL_cost_* results in the fastest lifts (Table 2), since cumulative load accumulates over time.

**Table 2.** Phase durations (in seconds) for all three phases for all optimal control problem (OCP) formulations

OCP	Human only				Human with exoskeleton			
	Phase 1 (s)	Phase 2 (s)	Phase 3 (s)	Total (s)	Phase 1 (s)	Phase 2 (s)	Phase 3 (s)	Total (s)
LSQ	1.34	0.12	1.42	2.88	1.34	0.12	1.42	2.88
*CLBL_cost_*	1.21	0.14	1.00	2.35	1.60	0.14	1.06	2.80
*PLBL_cost_*	1.22	0.14	1.19	2.55	1.60	0.14	1.31	3.04
*HYB_cost_*	1.25	0.14	1.19	2.58	1.53	0.14	1.10	2.77

*Abbreviations.* CLBL, cumulative low-back load; LSQ, least squares; PLBL, peak low-back load.

## Discussion

The risk of low-back injury can be reduced by improving lifting technique and by using an exoskeleton (Toxiri et al., 2019). Two of the biomechanic metrics that have been associated with the risk of injury to the lower back are CLBL and PLBL. In this study, we used simulation to examine how effective training, and an exoskeleton, are at reducing the CLBL and PLBL during a stoop–squat lift of a 10-kg box. We then compare the contributions of the improved technique alone, and with the exoskeleton assistance, to the HO reference motion that we have reconstructed from motion capture data. The cost functions we use do not only change the motion of the human in order to decrease the risk of injury, but they affect the way the exoskeleton supports the human as well, in order to help decrease the risk of injury.

**Figure A1.**
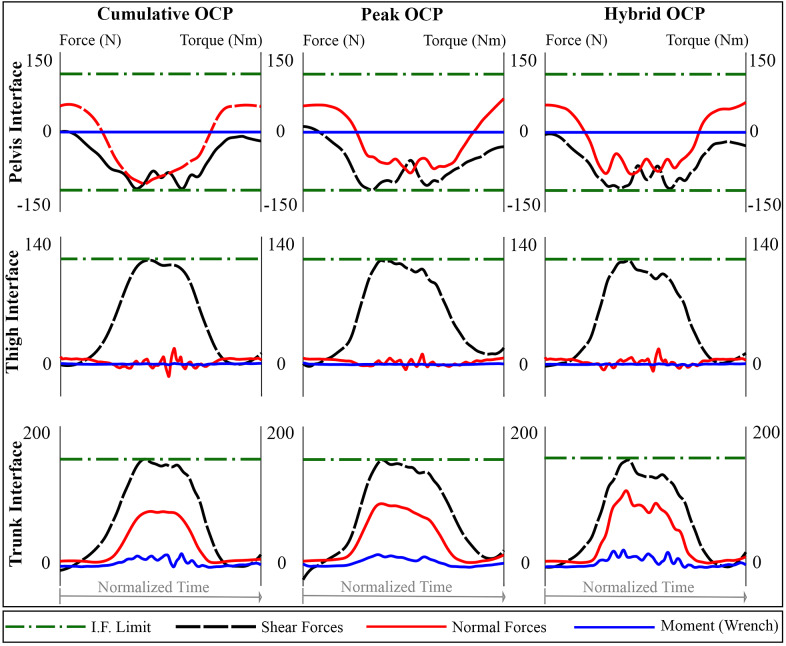
Interaction forces for human-with-exoskeleton prediction optimal control problems. Values shown correspond to normal and shear forces, and for moments about the three attachment points: pelvis, torso, and thigh. Interaction force limits are also reported.

We have had to make simplifications to our model, because the human body, the exoskeleton, and the interaction of the two are complex. We have ignored the effects of muscles and muscle dynamics. This simplification is reasonable under the assumption that the human is performing a submaximal lift and is not at the limits of the participant’s flexibility, force, or speed. By creating three hypothetical lifting techniques using *CLBL_cost_*, *PLBL_cost_*, and *HYB_cost_*, we assume that humans can actually be trained to minimize these risk factors. We have also included limits on the interaction forces (Figure A1 in Appendix A) under the assumption that the exoskeleton is comfortable to use if these limits are met. While we have imposed human–exoskeleton interaction force limits, it remains unclear how interaction forces will affect the motion of the human.

The HO L5/S1 peak moments compare well (176–210 Nm) to the ones reported by Kingma et al. (2004; 187–211 Nm; Figure 6a), as well as the peak lumbar flexion angles (Figure 7). In a study by Lavender et al. (2002), 265 participants were trained to perform a lift of a 12–13-kg box to improve their lifting technique. The peak moment varied from 225 to 178 Nm throughout the training period. In relation to our HO simulation of lowering the PLBL, we can see that this reduction is similar to the reduction in PLBL in our *PLBL_cost_* HO simulations, as the HO reference motion has a PLBL of 210 Nm and *PLBL_cost_* HO OCP minimizes this to 176 Nm. De Looze et al. (2016) report similar peak moment reductions of 19.5 and 15% when participants use the PLAD (Abdoli-E et al., 2006) and BNDR (Wehner et al., 2010) exoskeletons. In contrast, Koopman et al. (2020a) report a 5–10% reduction in peak moments when using the Laevo exoskeleton. In the context of active back-support exoskeletons, the XoTrunk exoskeleton (Lazzaroni et al., 2020) achieved a peak moment reduction of 17%. The passive SPEXOR exoskeleton produced a maximum of 23 ± 3% reduction in the peak L5/S1 extension moment (Koopman et al., 2020b) for 10 subjects using different lifting techniques, including exoskeleton assistance. Using our simulations based on the active SPEXOR exoskeleton, we predict a reduction of 21.4% (according to the CLBL cost function) to 34.3% (according to the PLBL cost function).

## Conclusion

Our simulations indicate that improving the lifting technique alone can reduce the CLBL from 243 to 130 Nms, but only modestly reduces the PLBL from 210 to 176 Nm. When the SPEXOR exoskeleton is used without any alteration to the person’s lifting technique, the CLBL is reduced from 243 to 217 Nms, while the PLBL is reduced from 210 to 177 Nm. The biggest reductions in both CLBL and PLBL are realized when both the SPEXOR exoskeleton and improved lifting technique are used together where the CLBL is reduced to 121 Nms and the PLBL to 138 Nm. While all of the three cost functions we examined reduced the risk factors for back injury, the HYB cost function offered the most balanced reduction of both the CLBL (38.3%) and the PLBL (28.6%). Thus, improvements to lifting technique alone may be a suitable intervention for people who infrequently lift light loads. However, in more demanding tasks, an exoskeleton should be considered, since it is more effective than the technique alone at reducing both the cumulative and peak loads experienced by the low back.

## Data Availability

The data that support the findings of this study are available from the corresponding author, G.M., upon reasonable request.

## References

[r1] Abdoli-E M , Agnew MJ and Stevenson JM (2006) An on-body personal lift augmentation device (PLAD) reduces EMG amplitude of erector spinae during lifting tasks. Clinical Biomechanics 21(5), 1–16. 16494978 10.1016/j.clinbiomech.2005.12.021

[r2] Baumgarte J (1972) Stabilization of constraints and integrals of motion in dynamical systems. Computer Methods in Applied Mechanics and Engineering 1(1), 1–16.

[r3] Bock HG and Plitt KJ (1985) Multiple shooting algorithm for direct solution of optimal control problems. IFAC Proceedings Series 17(2), 1603–1608.

[r4] Brereton LC and McGill SM (1999) Effects of physical fatigue and cognitive challenges on the potential for low back injury. Human Movement Science 18(6), 839–857.

[r5] Buchbinder R , Blyth FM , March LM , Brooks P , Woolf AD and Hoy DG (2013) Placing the global burden of low back pain in context. Best Practice and Research: Clinical Rheumatology 27(5), 575–589. 24315140 10.1016/j.berh.2013.10.007

[r6] Burgess-Limerick R , Abernethy B , Neal RJ and Kippers V (1995) Self-selected manual lifting technique: Functional consequences of the interjoint coordination. Human Factors 37(2), 395–411. 7642185 10.1518/001872095779064537

[r7] Christophy M , Senan NAF , Lotz JC and O’Reilly OM (2012) A musculoskeletal model for the lumbar spine. Biomechanics and Modeling in Mechanobiology 11(1–2), 19–34. 21318374 10.1007/s10237-011-0290-6

[r8] Coenen P , Kingma I , Boot CR , Bongers PM and Van Dieën JH (2012) The contribution of load magnitude and number of load cycles to cumulative low-back load estimations: A study based on in-vitro compression data. Clinical Biomechanics 27(10), 1083–1086. 22877832 10.1016/j.clinbiomech.2012.07.010

[r9] Coenen P , Kingma I , Boot CR , Bongers PM and Van Dieën JH (2014) Cumulative mechanical low-back load at work is a determinant of low-back pain. Occupational and Environmental Medicine 71(5), 332–337. 24676271 10.1136/oemed-2013-101862

[r10] Coenen P , Kingma I , Boot CR , Twisk JW , Bongers PM and Van Dieën JH (2013) Cumulative low back load at work as a risk factor of low back pain: A prospective cohort study. Journal of Occupational Rehabilitation 23(1), 11–18. 22718286 10.1007/s10926-012-9375-z PMC3563950

[r11] de Leva P (1996) Adjustments to Zatsiorsky–Seluyanov’s segment inertia parameters. Journal of Biomechanics 29(9), 1223–1230. 8872282 10.1016/0021-9290(95)00178-6

[r12] de Looze MP , Bosch T , Krause F , Stadler KS and O’Sullivan LW (2016) Exoskeletons for industrial application and their potential effects on physical work load. Ergonomics 59(5), 671–681. 26444053 10.1080/00140139.2015.1081988

[r13] de Zee M , Hansen L , Wong C , Rasmussen J and Simonsen EB (2007) A generic detailed rigid-body lumbar spine model. Journal of Biomechanics 40(6), 1219–1227. 16901492 10.1016/j.jbiomech.2006.05.030

[r14] Felis ML (2017) RBDL: An efficient rigid-body dynamics library using recursive algorithms. Autonomous Robots 41(2), 495–511.

[r15] M. Harant , M. Millard , N. Šarabon and K. Mombaur , “Cost function evaluation for optimizing design and actuation of an active exoskeleton to ergonomically assist lifting motions,” 2019 IEEE-RAS 19th International Conference on Humanoid Robots (Humanoids), 2019, pp. 186–193, doi: 10.1109/Humanoids43949.2019.9035028. in Toronto, Canada.

[r16] Hensel R and Keil M (2019) Subjective evaluation of a passive industrial exoskeleton for lower-back support: A field study in the automotive sector. IISE Transactions on Occupational Ergonomics and Human Factors 7(3–4), 213–221.

[r17] Holladay JC (1957) A smoothest curve approximation. American Mathematical Society: Mathematical Tables and Other Aids to Computation 11(60), 233–243.

[r18] Huysamen K , de Looze M , Bosch T , Ortiz J , Toxiri S and O’Sullivan LW (2018) Assessment of an active industrial exoskeleton to aid dynamic lifting and lowering manual handling tasks. Applied Ergonomics 68(Apr 2017), 125–131. 29409626 10.1016/j.apergo.2017.11.004

[r19] Jäger M , Jordan C , Theilmeier A , Wortmann N , Kuhn S , Nienhaus A and Luttmann A (2013) Lumbar-load analysis of manual patient-handling activities for biomechanical overload prevention among healthcare workers. Annals of Occupational Hygiene 57(4), 528–544. 23253360 10.1093/annhyg/mes088

[r20] Kazerooni H , Tung W and Pillai M (2019) Evaluation of trunk-supporting exoskeleton. Proceedings of the Human Factors and Ergonomics Society Annual Meeting 63(1), 1080–1083.

[r21] Kingma I , Bosch T , Bruins L and van Dieën JH (2004) Foot positioning instruction, initial vertical load position and lifting technique: Effects on low back loading. Ergonomics 47(13), 1365–1385. 15513714 10.1080/00140130410001714742

[r22] Koopman AS , Kingma I , de Looze MP and van Dieën JH (2020a) Effects of a passive back exoskeleton on the mechanical loading of the low-back during symmetric lifting. Journal of Biomechanics 102, 109486. 31718821 10.1016/j.jbiomech.2019.109486

[r23] Koopman AS , Näf M , Baltrusch SJ , Kingma I , Rodriguez-Guerrero C , Babič J , de Looze MP and van Dieën JH (2020b) Biomechanical evaluation of a new passive back support exoskeleton. Journal of Biomechanics 105, 109795. 32423541 10.1016/j.jbiomech.2020.109795

[r24] Laevo BV (2018) Product Information—What Is the Laevo and How Does It Work? Available at: http://en.laevo.nl (accessed January 2018).

[r25] Eric P. Lorenz , Steven A. Lavender & Gunnar B. J. Andersson (2002) Determining what should be taught during lift-training instruction, Physiotherapy Theory and Practice, 18:4, 175–191, DOI: 10.1080/09593980290058580.

[r26] Lazzaroni M , Tabasi A , Toxiri S , Caldwell DG , De Momi E , van Dijk W , de Looze MP , Kingma I , van Dieën JH and Ortiz J (2020) Evaluation of an acceleration-based assistive strategy to control a back-support exoskeleton for manual material handling. Wearable Technologies 1(May), 1–16. 10.1017/wtc.2020.8 PMC11265403 39050266

[r27] Leineweber DB , Bauer I , Bock HG and Schlöder JP (2003) An efficient multiple shooting based reduced SQP strategy for large-scale dynamic process optimization. Part 1: Theoretical aspects. Computers and Chemical Engineering 27(2), 157–166.

[r28] Z. Luo and Y. Yu , ”Wearable stooping-assist device in reducing risk of low back disorders during stooped work,” 2013 IEEE International Conference on Mechatronics and Automation, 2013, pp. 230–236, doi: 10.1109/ICMA.2013.6617923. in Takamatsu, Kagawa, Japan.

[r29] Marinou G.D. , Mombaur K.D. (2022) Optimizing Active Spinal Exoskeletons to Minimize Low Back Loads. In: Moreno J.C. , Masood J. , Schneider U. , Maufroy C. , Pons J.L. (eds) Wearable Robotics: Challenges and Trends. WeRob 2020. Biosystems & Biorobotics, vol 27. Springer, Cham. 10.1007/978-3-030-69547-7_73.

[r30] McGill SM and Norman RW (1987) Effects of an anatomically detailed erector spinae model on L4L5 disc compression and shear. Journal of Biomechanics 20(6), 591–600. 3611135 10.1016/0021-9290(87)90280-6

[r31] Millard M , Sreenivasa M and Mombaur K (2017) Predicting the motions and forces of wearable robotic systems using optimal control. Frontiers Robotics AI 4(Aug), 1–12.

[r32] Mitsuhashi N , Fujieda K , Tamura T , Kawamoto S , Takagi T and Okubo K (2009) BodyParts3D: 3D structure database for anatomical concepts. Nucleic Acids Research 37(Suppl. 1), 782–785. 10.1093/nar/gkn613 PMC2686534 18835852

[r33] Muramatsu Y and Kobayashi H (2014) Assessment of local muscle fatigue by NIRS—development and evaluation of muscle suit. ROBOMECH Journal 1(1), 1–11.

[r34] Näf MB , Koopman AS , Baltrusch S , Rodriguez-Guerrero C , Vanderborght B and Lefeber D (2018) Passive back support exoskeleton improves range of motion using flexible beams. Frontiers Robotics AI 5(June), 1–16. 10.3389/frobt.2018.00072 PMC7805753 33500951

[r35] Nagarajan V and Kolter JZ (2017) Gradient descent GAN optimization is locally stable. In Proceeding of the Advances in Neural Information Processing Systems, pp. 5586–5596.

[r36] Pearcy MJ and Bogduk N (1988) Instantaneous axes of rotation of the lumbar intervertebral joints. Spine 13(9), 1033–1041. 3206297 10.1097/00007632-198809000-00011

[r37] Toxiri S , Koopman AS , Lazzaroni M , Ortiz J , Power V , de Looze MP , O’Sullivan L and Caldwell DG (2018) Rationale, implementation and evaluation of assistive strategies for an active back-support exoskeleton. Frontiers Robotics AI 5(May), 1–14. 10.3389/frobt.2018.00053 PMC7805873 33500935

[r38] Toxiri S , Näf MB , Lazzaroni M , Fernández J , Sposito M , Poliero T , Monica L , Anastasi S , Caldwell DG and Ortiz J (2019) Back-support exoskeletons for occupational use: An overview of technological advances and trends. IISE Transactions on Occupational Ergonomics and Human Factors 7(3–4), 237–249.

[r39] Ulrey BL and Fathallah FA (2013) Effect of a personal weight transfer device on muscle activities and joint flexions in the stooped posture. Journal of Electromyography and Kinesiology 23(1), 195–205. 23021604 10.1016/j.jelekin.2012.08.014

[r40] van Dieën JH (2005) Effects of antagonistic co-contraction on differences between electromyography based and optimization based estimates of spinal forces. Ergonomics 48(4), 411–426. 15804849 10.1080/00140130512331332918

[r41] Wehner, M , Rempel, D , & Kazerooni, H . “Lower Extremity Exoskeleton Reduces Back Forces in Lifting.” Proceedings of the ASME 2009 Dynamic Systems and Control Conference. ASME 2009 Dynamic Systems and Control Conference, Volume 2. Hollywood, California, USA. October 12–14, 2009. pp. 49-56. ASME. 10.1115/DSCC2009-2644.

[r42] Wong KW , Luk KD , Leong JC , Wong SF and Wong KK (2006) Continuous dynamic spinal motion analysis. Spine 31(4), 414–419. 16481951 10.1097/01.brs.0000199955.87517.82

[r43] Wynne-Jones G , Cowen J , Jordan JL , Uthman O , Main CJ , Glozier N and Van Der Windt D (2014) Absence from work and return to work in people with back pain: A systematic review and meta-analysis. Occupational and Environmental Medicine 71(6), 448–458. 24186944 10.1136/oemed-2013-101571 PMC4033140

[r44] Xiang Y and Arefeen A (2020) Two-dimensional team lifting prediction with floating-base box dynamics and grasping force coupling. Multibody System Dynamics 50(2), 211–231.

